# How the methodology of 3D structure preparation influences the quality of QSPR models?

**DOI:** 10.1186/1758-2946-4-S1-P61

**Published:** 2012-05-01

**Authors:** Stanislav Geidl, Roman Beránek, Radka Svobodová Vařeková, Tomáš Bouchal, Miroslav Brumovský, Michal Kudera, Ondřej Skřehota, Jaroslav Koča

**Affiliations:** 1 National Centre for Biomolecular Research and CEITEC - Central European Institute of Technology, Masaryk University, Brno, Czech Republic, 625 00, CZ

## 

QSPR modelling is a very useful and popular methodology for estimating the physical and chemical properties of molecules. The inputs for QSPR models are 3D structures of molecules. Currently, the 3D structures for millions of molecules are publicly available. A large number of these 3D structures were generated by software tools for the conversion of 2D structures into 3D. Moreover, the generated structures can be geometrically optimized by different approaches, such as molecular mechanics, quantum mechanics, etc.. The question arises as to how the methodology of 3D structure preparation influences the quality of QSPR models that use these structures. Is there some software tool for 3D structure construction more suitable for QSPR modelling purposes than others? Conversely, which software tools are inappropriate? How strong is the influence of the geometry optimization procedure? We focused on these questions in the present study.

In our work, we analyzed the influence of 3D structure preparation methodology on the quality of QSPR models for the prediction of the acid dissociation constant (pK_a_) from atomic charges [1]. We employed three different software tools for 3D structure generation (Corina [2], Balloon [3], etc.), together with two approaches for geometry optimization. This way, we prepared nine sets of 3D structures, and used them to develop QSPR models based on several different charge calculation schemes. Afterwards, we compared the accuracy of these QSPR models and discussed the influence of the methodology for 3D structure preparation.
